# Non-contiguous finished genome sequence and description of *Kallipyga massiliensis* gen. nov., sp. nov., a new member of the family *Clostridiales* *Incertae Sedis* *XI*

**DOI:** 10.4056/sigs.4047997

**Published:** 2013-07-30

**Authors:** Perrine Hugon, Dhamodharan Ramasamy, Catherine Robert, Carine Couderc, Didier Raoult, Pierre-Edouard Fournier

**Affiliations:** 1 Aix-Marseille Université, Faculté de médecine, Marseille, France

**Keywords:** *Kallipyga massiliensis*, genome, culturomics, taxonogenomics

## Abstract

*Kallipyga massiliensis* strain ph2^T^ is the type strain of *Kallipyga massiliensis* gen. nov., sp. nov., the type species of the new genus *Kallipyga* within the family *Clostridiales Incertae Sedis XI.* This strain, whose genome is described here, was isolated from the fecal flora of a 26-year-old woman suffering from morbid obesity. *K. massiliensis* is an obligate anaerobic coccus. Here we describe the features of this organism, together with the complete genome sequence and annotation. The 1,770,679 bp long genome (1 chromosome but no plasmid) contains 1,575 protein-coding and 50 RNA genes, including 4 rRNA genes.

## Introduction

*Kallipyga massiliensis* strain ph2^T^ (CSUR=P241, DSM=26229) is the type strain of *K. massiliensis* gen. nov., sp. nov. This bacterium was isolated from the stool sample of an obese French patient as part of a study aiming at individually cultivating all species occurring within human feces [1-3]. It is a Gram-positive, anaerobic, indole-negative coccus. Defining the taxonomic status of bacterial isolates remains a challenging task. The taxonomic molecular tools currently available, including 16S rRNA sequence similarity, G + C content and DNA–DNA hybridization (DDH) [4,5], although considered as gold standards, have limitations [6,7]. The 16S rRNA sequence similarity and G+C content thresholds do not apply uniformly to all species or genera, and the DDH method lacks intra- and inter-laboratory reproducibility [5]. The advent of high-throughput genome sequencing and proteomic analysis [8] has granted unprecedented access to exhaustive genetic and protein information for bacterial isolates. We recently proposed a polyphasic approach to describe new bacterial species in which genome sequences and MALDI-TOF spectra are used along with phenotypic characteristics [9-30].

The family *Clostridiales* *Incertae Sedis* XI (Garrity and Holt 2001) was created in 2001 [31] and currently includes the 11 following genera: *Anaerococcus* (Ezaki *et al*. 2001) [32], *Dethiosulfatibacter* (Takii *et al*. 2007) [33], *Finegoldia* (Murdoch and Shah 2000) [34], *Gallicola* (Ezaki *et al*. 2001) [32], *Helcococcus* (Collins *et al*. 1993) [35], *Parvimonas* (Tindall and Euzéby 2006) [36], *Peptoniphilus* (Ezaki *et al*. 2001) [32], *Sedimentibacter* (Breitenstein *et al*. 2002) [37], *Soehngenia* (Parshina *et al*. 2003) [38], *Sporanaerobacter* (Hernandez-Eugenio *et al*. 2002) [39] and *Tissierella* (Collins and Shah 1986) [40]. Currently, 31 species with validly published names are reported in this family [41]. The species listed in the *Clostridiales* *Incertae Sedis* XI are mostly comprised of Gram-positive, obligate anaerobic cocci. Members belonging to this family were identified as pathogens in both humans and animals. In humans, they were often isolated from patients with septic arthritis, necrotizing pneumonia, prosthetic joint infection and other clinical conditions associated with vaginal discharges and ovarian, peritoneal and sacral abscesses [42-46].

Here we present a summary classification and a set of features for *K. massiliensis* gen. nov., sp. nov., strain ph2^T^ (CSUR=P241, DSM=26229) together with the description of the complete genomic sequencing and annotation. These characteristics support the circumscription of the genus *Kallipyga* and its type species, *K. massiliensis* within the *Clostridiales* *Incertae Sedis* XI family.

## Classification and features

**Figure 1:**
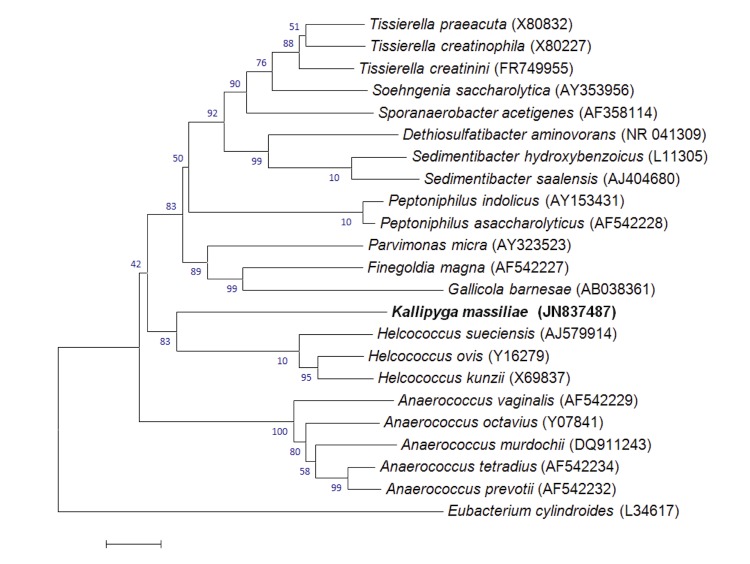
Phylogenetic tree highlighting the position of *Kallipyga massiliensis* strain ph2^T^ relative to other type strains within the *Clostridiales* *Incertae Sedis* XI family. Genbank accession numbers are indicated in parentheses. Sequences were aligned using CLUSTALW, and phylogenetic inferences obtained using the maximum-likelihood method in MEGA software. Numbers at the nodes are percentages of bootstrap values obtained by repeating the analysis 500 times to generate a majority consensus tree. *Eubacterium cylindroides* was used as outgroup. The scale bar represents a 2% nucleotide sequence divergence.

A stool sample was collected from a 26-year-old woman living in Marseille (France). She suffered from morbid obesity and had a body mass index of 48.2 (118.8 kg, 1.57 meter). At the time of stool sample collection she did not take any medication and was not on a diet. The patient gave an informed and signed consent, and the agreement of the ethics committee of the Institut Fédératif de Recherche (IFR48, Faculty of Medicine, Marseille, France) was obtained under reference 09-022. Another four new bacterial species, *Alistipes obesi*, *Peptoniphilus grossensis*, *P. obesi* and *Enorma massiliensis* [25-27,33], were also isolated from this specimen using various culture conditions. The fecal specimen was preserved at -80°C after collection. Strain ph2 ^T^ (Table 1) was isolated in 2011 by anaerobic culture on 5% sheep blood-enriched agar in anaerobic atmosphere at 37°C, following 26 days in a blood culture bottle with rumen and sheep blood. The 16S rRNA nucleotide sequence (GenBank accession number JN837487) of *Kallipyga massiliensis* strain ph2^T^ was 86.09% similar to *Helcococcus sueciensis*, the phylogenetically closest species (Figure 1). This value was lower than the 95.0% 16S rRNA gene sequence threshold recommended by Stackebrandt and Ebers (2006) to delineate a new genus without carrying out DNA-DNA hybridization [5].

**Table 1:** Classification and general features of *Kallipyga massiliensis* strain ph2^T^ according to the MIGS recommendations [45].

**MIGS ID**	**Property**	**Term**	**Evidence code^a^**
		Domain *Bacteria*	TAS [47]
		Phylum *Firmicutes*	TAS [48-50]
		Class *Clostridia*	TAS [51,52]
	Current classification	Order *Clostridiales*	TAS [53,54]
		Family *Clostridiales* *Incertae Sedis* XI	TAS [55]
		Genus *Kallipyga*	IDA
		Species *Kallipyga massiliensis*	IDA
		Type strain ph2^T^	IDA
	Gram stain	Positive	IDA
	Cell shape	Cocci	IDA
	Motility	Non-motile	IDA
	Sporulation	Non-sporulating	IDA
	Temperature range	Mesophile	IDA
	Optimum temperature	37°C	IDA
MIGS-6.3	Salinity	unknown	IDA
MIGS-22	Oxygen requirement	Anaerobic	IDA
	Carbon source	Unknown	NAS
	Energy source	Unknown	NAS
MIGS-6	Habitat	Human gut	IDA
MIGS-15	Biotic relationship	Free living	IDA
MIGS-14	Pathogenicity Biosafety level Isolation	Unknown 2 Human feces	NAS
MIGS-4	Geographic location	France	IDA
MIGS-5	Sample collection time	January 2011	IDA
MIGS-4.1	Latitude	43.296482	IDA
MIGS-4.1	Longitude	5.36978	IDA
MIGS-4.3	Depth	Surface	IDA
MIGS-4.4	Altitude	0 m above sea level	IDA

Evidence codes - IDA: Inferred from Direct Assay; TAS: Traceable Author Statement (i.e., a direct report exists in the literature); NAS: Non-traceable Author Statement (i.e., not directly observed for the living, isolated sample, but based on a generally accepted property for the species, or anecdotal evidence). These evidence codes are from the Gene Ontology project [56]. If the evidence is IDA, then the property was directly observed for a live isolate by one of the authors or an expert mentioned in the acknowledgements.

By comparison to the Genbank database [57], strain ph2^T^ also exhibited a nucleotide sequence similarity greater than 98.7% with 16 sequences from uncultured bacteria from the human skin microbiome [58]. These bacteria are most likely classified within the same species as strain ph2^T^.

**Figure 2:**
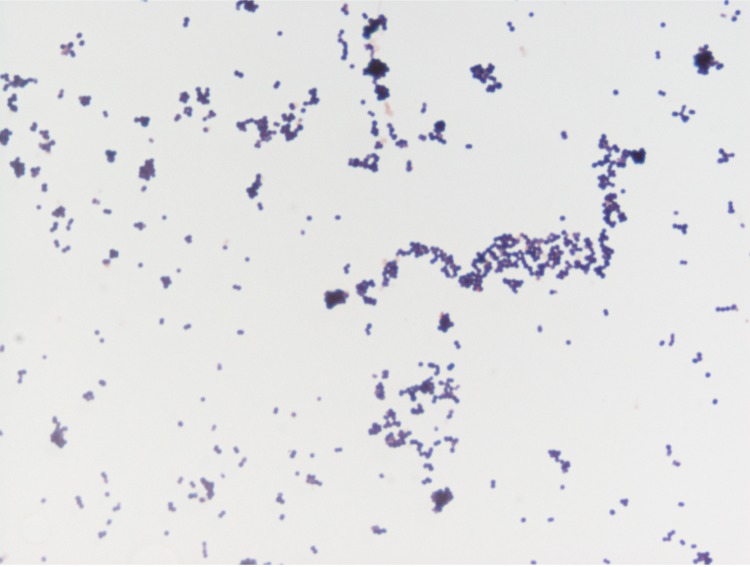
Gram staining of *K massiliensis* strain ph2^T^

**Figure 3:**
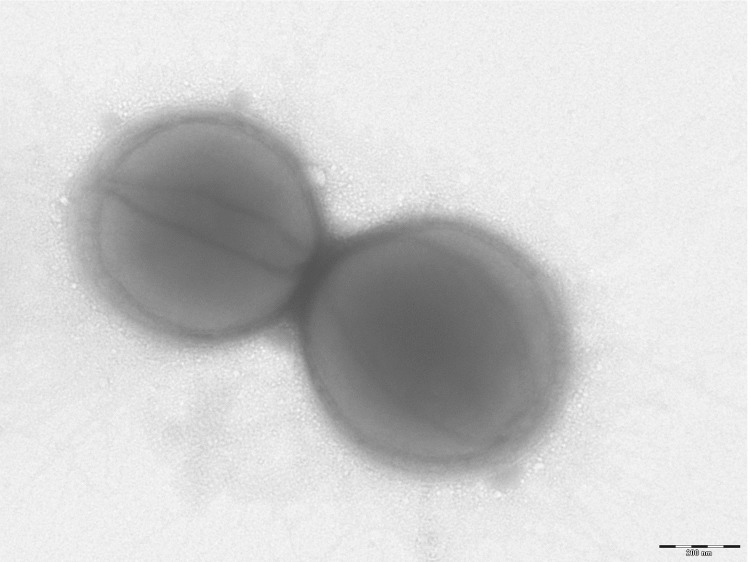
Transmission electron microscopy of *K. massiliensis* strain ph2^T^ using a Morgani 268D (Philips) at an operating voltage of 60kV. The scale bar represents 200 nm.

Different growth temperatures (25, 30, 37, 45°C) were tested; no growth occurred at 25°C and 30°C, growth occurred between 37°C and 45°C, and optimal growth was observed at 37°C. Colonies were bright grey with a diameter of 1.0 mm on 5% blood-enriched Columbia agar. Growth of the strain was tested under anaerobic and microaerophilic conditions using GENbag anaer and GENbag microaer systems, respectively (BioMérieux), and in the presence of air, with or without 5% CO_2_. Optimal growth was obtained anaerobically. No growth was observed under aerobic and microaerophilic conditions. Gram staining showed Gram-positive cocci (Figure 2). A motility test was negative. Cells grown on agar are Gram-positive, have a diameter in electron microscopy ranging from 0.57µm to 0.78µm (mean, 0.67 µm, Figure 3) and are mostly grouped in pairs, short chains or small clumps.

Strain ph2^T^ exhibited neither catalase or oxidase activities. Using API 32A (BioMerieux), nitrate reduction, indole formation and urease production were negative. A positive reaction was obtained for α-galactosidase, arginine dihydrolase and arginine arylamidase, α-glucosidase and β-glucosidase. Strain ph2^T^ did not ferment mannose or raffinose. Negative reactions were observed for β-galactosidase, β-galactosidase-6-phosphate, α-arabinosidase, β-glucuronidase, N-acetyl-β-glucosaminidase, glutamic acid decarboxylase, proline arylamidase, leucyl glycine arylamidase, phenylalanine arylamidase, pyroglutamic acid arylamidase, tyrosine arylamidase, alanine arylamidase, glycine arylamidase, histidine arylamidase, glutamyl glutamic acid arylamidase, and serine arylamidase. Using an API Zym (BioMerieux), positive reactions were observed for esterase lipase, leucine arylamidase, α-glucosidase, β-glucosidase and acid phosphatase. Negative reactions were obtained for esterase, lipase, valine and cysteine arylamidase, trypsine, α-chymotrypsine, naphthol-AS-BI-phosphohydrolase, α-galactosidase, β-galactosidase, β-glucuronidase, N-acetyl-β-glucosaminidase, α-mannosidase and α-fucosidase. Using an API 50CH (BioMerieux), *K. massiliensis* weakly fermented D-ribose, D-glucose, D-fructose and aesculin. By comparison with its closest phylogenetic neighbors, *K. massiliensis* differed from *Finegoldia magna* in α-galactosidase and α-glucosidase production, D-ribose and D-fructose fermentation. It also differed from *Helcococcus kunzii* in oxygen requirement, α-galactosidase and leucine arylamidase production, D-ribose, D-glucose and esculin utilization. It differed from *Parvimonas micra* in alkaline phosphatase, glutamyl glutamic acid arylamidase, β-glucosidase, phenylalanine arylamidase and histidine arylamidase production and D-glucose fermentation. It differed from *Peptoniphilus indolicus* in α-galactosidase, indole, α-glucosidase, β-glucosidase, dihydrolase phenylalanine, phenylalanine arylamidase and histidine arylamidase production, and D-glucose fermentation (Table 2).

**Table 2:** Differential characteristics of *Kallipyga massiliensis* gen. nov., sp. nov., strain ph2^T^, *Finegoldia magna* strain ATCC 29328, *Helcococcus kunzii* strain ATCC 51366, *Parvimonas micra* strain ATCC 33270 and *Peptoniphilus indolicus* strain ATCC 29427^T^.

**Properties**	*K. massiliensis*	*F. magna*	*H. kunzii*	*P. micra*	*P. indolicus*
Cell diameter (µm)	0.67	na	na	0.3-0.7	na
Oxygen requirement	anaerobic	anaerobic	facultative anaerobic	anaerobic	anaerobic
Colony color	bright gray	var		na	na
Gram stain	+	+	+	+	+
Salt requirement	-	na	+/-	na	-
Motility	-	-	-	na	-
Endospore formation	-	-	na	na	-
**Production of**					
Alkaline Phosphatase	-	+/-	-	+	-
Catalase	-	+/-	-	na	na
Oxidase	-	na	na	na	na
Nitrate reductase	-	-	-	na	na
Urease	-	-	na	na	-
α-galactosidase	+	-	-	na	-
β-galactosidase	-	-	-	na	-
Indole	-	-	na	-	+
Arginine arylamidase	+	+	na	+	+
Glutamyl glutamic acid arylamidase	-	na	na	+	na
Arginine dihydrolase	+	+/-	na	na	-
α-glucosidase	+	-	na	na	-
β-glucosidase	+	na	na	-	-
β-glucuronidase	-	-	-	na	na
Phenylalanine arylamidase	-	-	na	+	+
Esterase lipase	+	na	na	na	na
Leucine arylamidase	+	+	-	na	+
Cystine arylamidase	-	na	na	na	na
Histidine arylamidase	-	-/w	na	+	+
**Fermentation of**					
D-mannose	-	-	na	na	-
D-ribose	w	-	-	na	na
D-glucose	w	-/w	-	-	-
D-fructose	w	+	na	na	na
Esculin	w	na	+	na	na
**Isolated from**	human gut	human	human	human	mastitis of cattle

na = data not available; var = variable; w = weak

*K. massiliensis* is susceptible to amoxicillin, amoxicillin-clavulanic acid, gentamicin 500, penicillin, imipenem, vancomycin, rifampicin and nitrofurantoin, but resistant to ciprofloxacin, metronidazole, gentamicin 10, trimethoprim/sulfamethoxazole, ceftriaxon, erythromycin and doxycycline.

**Figure 4:**
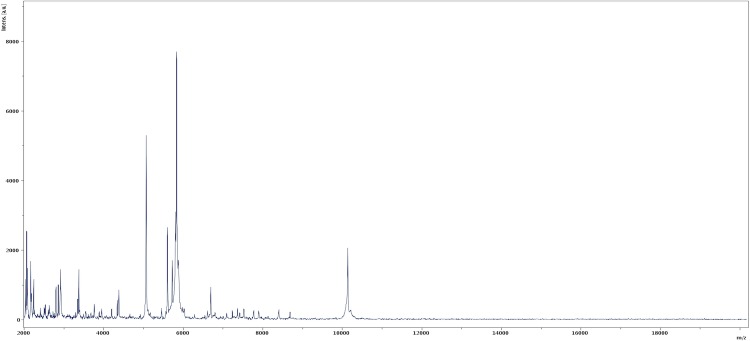
Reference mass spectrum from *K. massiliensis* strain ph2^T^. Spectra from 12 individual colonies were compared and a reference spectrum was generated.

**Figure 5:**
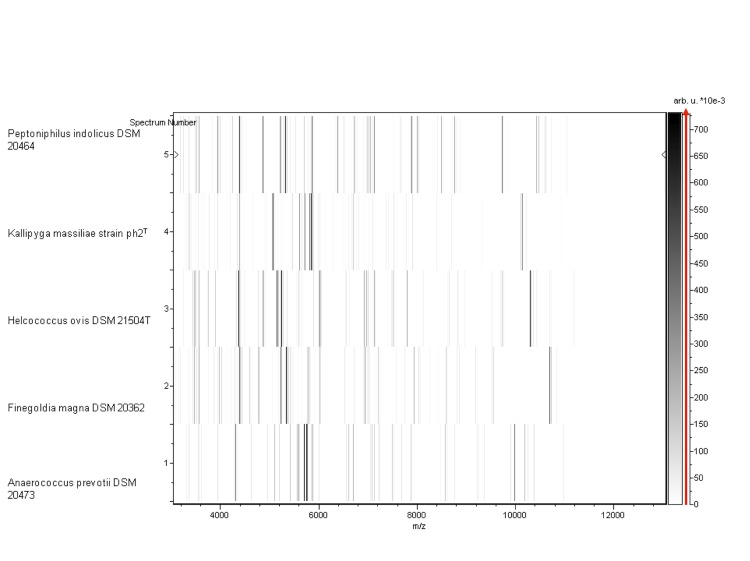
Gel view comparing *Kallipyga massiliensis* sp. nov strain ph2^T^ to other phylogenetically close species. The gel view displays the raw spectra of loaded spectrum files arranged in a pseudo-gel like look. The x-axis records the m/z value. The left y-axis displays the running spectrum number originating from subsequent spectra loading. The peak intensity is expressed by a Gray scale scheme code. The color bar and the right y-axis indicate the relation between the color a peak is displayed with and the peak intensity in arbitrary units. Displayed species are indicated on the left.

Matrix-assisted laser-desorption/ionization time-of-flight (MALDI-TOF) MS protein analysis was carried out as previously described [59] using a Microflex spectrometer (Bruker Daltonics, Germany). Twelve distinct deposits were done for strain ph2^T^ from 12 isolated colonies. The twelve ph2^T^ spectra were imported into our database and compared to spectra from 3,769 bacteria using the MALDI BioTyper software (version 2.0, Bruker). A score enabled the presumptive identification and discrimination of the tested species from those in a database: a score > 2 with a validly published species enabled the identification at the species level; a score > 1.7 but < 2 enabled the identification at the genus level; and a score < 1.7 did not enable any identification. For strain ph2^T^, no significant score was obtained, suggesting that our isolate was not a member of any known species or genus (Figures 4 and 5). A broader study incorporating MALDI-TOF and 16S rDNA and genomic DNA identity data may be conducted to define taxonomic criteria at the family level.

## Genome sequencing information

### Genome project history

The organism was selected for sequencing on the basis of its phylogenetic position and 16S rRNA similarity to members of the family *Clostridiales* *Incertae Sedis* XI and is part of a study of the human digestive flora aiming at isolating all bacterial species within human feces [1-3]. It was the thirty-sixth genome from the family *Clostridiales Incertae Sedis* XI to be sequenced and the first genome of *K. massiliensis* gen. nov., sp. nov. The GenBank accession number is CAHC00000000 and consists of 22 contigs. Table 3 shows the project information and its association with MIGS version 2.0 compliance [60].

**Table 3:** Project information

**MIGS ID**	Property	Term
MIGS-31	Finishing quality	High-quality draft
MIGS-28	Libraries used	454 GS paired-end 3- kb libraries
MIGS-29	Sequencing platform	454 GS FLX Titanium
MIGS-31.2	Sequencing coverage	51.23 ×
MIGS-30	Assemblers	Newbler
MIGS-32	Gene calling method	Prodigal
	Genbank Date of Release	May 30, 2012
	NCBI project ID	CAHC00000000
MIGS-13	Project relevance	Study of the human gut microbiome

### Growth conditions and DNA isolation

*Kallipyga massiliensis* gen. nov., sp. nov., strain ph2^T^ (CSUR= P241, DSM=26229) was grown anaerobically on 5% sheep blood-enriched Columbia agar at 37°C. Three petri dishes were spread and the bacteria cultivated were resuspended in 3 × 100µl of G2 buffer (EZ1 DNA Tissue kit, Qiagen). A first mechanical lysis was performed by glass powder on the Fastprep-24 device (MP Biomedicals, USA) using 2 × 20 seconds cycles. DNA was then treated with 2.5µg/µL lysozyme for 30 minutes at 37°C and extracted using the BioRobot EZ1 Advanced XL (Qiagen). The DNA was then concentrated and purified on a QIAamp kit (Qiagen). The yield and concentration was measured by the Quant-it Picogreen kit (Invitrogen) on the Genios Tecan fluorometer at 78.2ng/µl.

### Genome sequencing and assembly

DNA (5 µg) was mechanically fragmented on a Hydroshear device (Digilab, Holliston, MA, USA) with an enrichment size at 3-4kb. The DNA fragmentation was visualized through the Agilent 2100 BioAnalyzer on a DNA labchip 7500 with an optimal size of 3.179kb. A 3kb paired-end library was constructed according to the 454 GS FLX Titanium paired-end protocol (Roche). Circularization and nebulization were performed and generated a pattern with an optimal at 600 bp. After PCR amplification through 17 cycles followed by double size selection, the single stranded paired-end library was quantified on the Quant-it Ribogreen kit (Invitrogen) on the Genios Tecan fluorometer at 58 pg/µL. The library concentration equivalence was calculated as 1.77E+08 molecules/µL. The library was stored at -20°C until further use.

The paired-end library was clonally amplified with 0.5 cpb and 1 cbp in 2 SV-emPCR reactions with the GS Titanium SV emPCR Kit (Lib-L) v2 (Roche). The yields of the emPCR were essentially the same at 12.3 and 12%, in the range of 5 to 20% recommended by the Roche procedure.

Approximately 790,000 beads were loaded on 1/4 region of a GS Titanium PicoTiterPlate PTP Kit 70x75 and sequenced with the GS FLX Titanium Sequencing Kit XLR70 (Roche). The run was performed overnight and then analyzed on the cluster through the gsRunBrowser and gsAssembler (Roche). A total, of 261,794 passed filter wells were obtained and generated 90.68 Mb with a length average of 346 bp. The global passed filter sequences were assembled using Newbler with 90% identity and 40 bp as overlap. The final assembly identified 3 scaffolds and 22 large contigs (> 1,500 bp) generating a genome size of 1.77 Mb which corresponds to a coverage of 51.23× genome equivalent.

### Genome annotation

Open Reading Frames (ORFs) were predicted using Prodigal [61] with default parameters. However, the predicted ORFs were excluded if they spanned a sequencing gap region. The predicted bacterial protein sequences were searched against the GenBank [57] and Clusters of Orthologous Groups (COG) databases using BLASTP. The tRNAs and rRNAs were predicted using the tRNAScan-SE [62] and RNAmmer [63] tools, respectively. Lipoprotein signal peptides and numbers of transmembrane helices were predicted using SignalP [64] and TMHMM [65], respectively. ORFans were identified if their BLASTP *E*-value was lower than 1e-03 for alignment length greater than 80 amino acids. If alignment lengths were smaller than 80 amino acids, we used an *E*-value of 1e-05. Such parameter thresholds have already been used in previous works to define ORFans. Artemis [66] and DNA Plotter [67] were used for data management and visualization of genomic features, respectively. Mauve alignment tool (version 2.3.1) was used for multiple genomic sequence alignment [68]. To estimate the mean level of nucleotide sequence similarity at the genome level between *K. massiliensis* and four other members of the family *Clostridiales* *Incertae Sedis* XI (Table 6), orthologous proteins were detected using the Proteinortho software [69] and genomes compared two by two. For each pair of genomes, we determined the mean percentage of nucleotide sequence identity among orthologous ORFs using BLASTn.

## Genome properties

**Figure 6:**
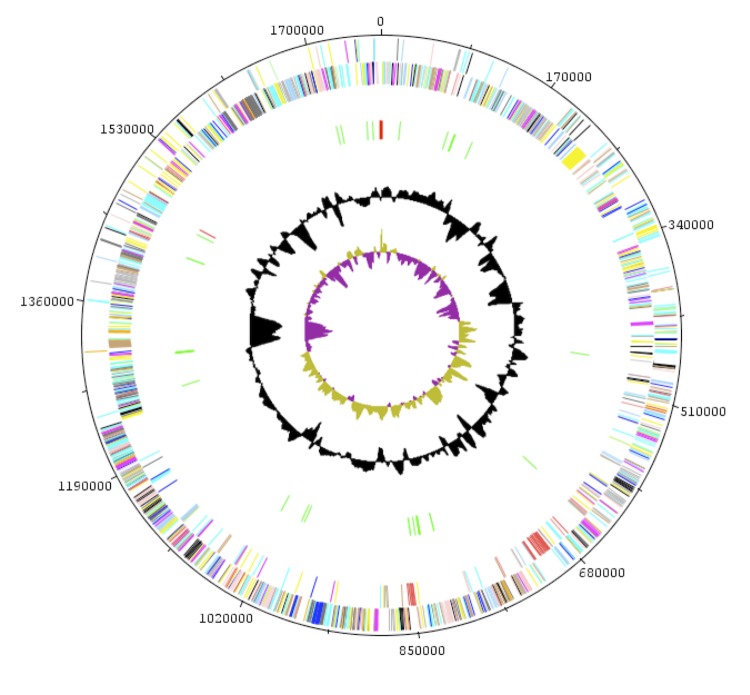
Graphical circular map of the chromosome. From the outside in, the outer two circles show the genes on the forward and reverse directions (colored by COG categories). The third circle marks the rRNA operon in red and tRNA genes in green. The fourth circle shows the G+C% content plot. The inner-most circle shows GC skew, with purple and olive indicating negative and positive values, respectively.

The genome is 1,770,679 bp long (one chromosome, no plasmid) with a G+C content of 51.40% (Figure 6 and Table 4). Of the 1,625 predicted chromosomal genes, 1,575 were protein-coding genes and 50 were RNAs. A total of 1,238 genes (76.18%) were assigned a putative function. Forty-two genes were identified as ORFans (2.66%) and the remaining genes were annotated as hypothetical proteins. The properties and statistics of the genome are summarized in Tables 4 and 5. The distribution of genes into COGs functional categories is presented in Table 5.

**Table 4:** Nucleotide content and gene count levels of the chromosome

**Attribute**	Value	% of total^a^
Genome size (bp)	1,770,679	
DNA coding region (bp)	1,590,528	89.82
DNA G+C content (bp)	910,129	51.40
Total genes	1,625	100
RNA genes	50	3.07
Protein-coding genes	1,575	96.92
Genes with function prediction	1,238	76.18
Genes assigned to COGs	1,165	71.69
Genes with peptide signals	90	5.53
Genes with transmembrane helices	405	24.92

^a^ The total is based on either the size of the genome in base pairs or the total number of protein-coding genes in the annotated genome

**Table 5:** Number of genes associated with the 25 general COG functional categories

**Code**	**Value**	**% age**^a^	**Description**
J	131	4.86	Translation
A	0	0.032	RNA processing and modification
K	75	5.31	Transcription
L	107	5.74	Replication, recombination and repair
B	0	0	Chromatin structure and dynamics
D	15	0.78	Cell cycle control, mitosis and meiosis
Y	0	0	Nuclear structure
V	51	1.53	Defense mechanisms
T	22	1.69	Signal transduction mechanisms
M	72	3.42	Cell wall/membrane biogenesis
N	1	0	Cell motility
Z	0	0	Cytoskeleton
W	0	0	Extracellular structures
U	13	0.84	Intracellular trafficking and secretion
O	45	2.47	Posttranslational modification, protein turnover, chaperones
C	66	4.53	Energy production and conversion
G	88	2.87	Carbohydrate transport and metabolism
E	67	6.16	Amino acid transport and metabolism
F	47	2.05	Nucleotide transport and metabolism
H	34	2.34	Coenzyme transport and metabolism
I	28	4.01	Lipid transport and metabolism
P	59	4.14	Inorganic ion transport and metabolism
Q	4	0.81	Secondary metabolites biosynthesis, transport and catabolism
R	127	8.15	General function prediction only
S	113	5.93	Function unknown
-	410	26.03	Not in COGs

^a^ The total is based on the total number of protein-coding genes in the annotated genome

## Genomic comparison of *K. massiliensis* and other members of the family *Clostridiales* *Incertae Sedis* XI.

Currently, 35 genomes are available for members of the family *Clostridiales* *Incertae Sedis* XI. Here, we compared the genome sequence of *K. massiliensis* strain ph2^T^ with those of *Finegoldia magna* strain ATCC 29328, *Helcococcus kunzii* strain ATCC 51366, *Peptoniphilus indolicus* strain ATCC 29427 and *Parvimonas micra* strain ATCC 33270. The draft genome of *K. massiliensis* (1.77Mb) is smaller than all other genomes except *P. micra* (1.70Mb) and exhibits a higher G+C content (51.40) (Table 6A). The gene content of *K. massiliensis* is also lower than the other four genomes used for comparison (Table 6B). In addition, *K. massiliensis* shared 653, 549, 592 and 548 orthologous genes with F*.magna, H. kunzii*,*P. indolicus* and *P.micra* respectively. The average nucleotide sequence identity ranged from 58.61 to 69.17% among *Clostridiales Incertae Sedis XI family* species, and from 58.61 to 59.97% between *K. massiliensis* and other species, thus confirming its new genus status (Table 6B).

**Table 6A:** Genomic comparison of *K. massiliensis* gen. nov., sp. nov., strain ph2^T^ with four other members of the family *Clostridiales* *Incertae Sedis* XI^†^

**Species**	**Strain**	**Genome accession number**	**Genome size (Mb)**	**G+C content**
*K. massiliensis*	ph2^T^	CAHC00000000	1,770,679	51.40
*F. magna*	ATCC 29328	NC_010376	1,797,577	32.1
*H. kunzii*	ATCC 51366	AGEI01000000	2,083,191	29.40
*P. indolicus*	ATCC 29427	AGBB01000000	2,101,630	31.70
*P. micra*	ATCC 33270	ABEE02000000	1,703,772	28.70

^†^Species and strain names, GenBank genome accession numbers, sizes and G+C contents.

**Table 6B:** Genomic comparison of *K. massiliensis* gen. nov., sp. nov., strain ph2^T^ with four other members of the family *Clostridiales* *Incertae Sedis* XI^†^

	*K. massiliensis*	*F. magna*	*H. kunzii*	*P. indolicus*	*P. micra*
*K. massiliensis*	**1,568**	635	549	592	548
*F. magna*	59.22	**1,656**	629	687	665
*H. kunzii*	59.06	68.20	**1,878**	561	560
*P. indolicus*	59.97	67.98	67.57	**2,205**	615
*P. micra*	58.61	69.17	68.52	68.64	**1,597**

**^†^** Numbers of orthologous proteins shared between genomes (upper right), average percentage of nucleotide similarity of orthologous proteins shared between genomes (lower left). Bold numbers indicate numbers of proteins per genome.

## Conclusion

On the basis of phenotypic, phylogenetic and genomic analyses, we formally propose the creation of *Kallipya massiliensis gen. nov.,* sp. nov., that contains the strain ph2^T^. This bacterium has been found in France.

### Description of *Kallipyga gen. nov.*

*Kallipyga* (cal.li.pi’ga N.L. fem. N. *Kallipyga* of the Greek epithet *kallipygos*, said of a statue of Aphrodite having beautifully proportioned buttocks).

Gram-positive cocci. Strictly anaerobic. Mesophilic. Non-Motile. Does not exhibit catalase, oxidase and indole production nor nitrate reduction. Positive for α-galactosidase, arginine dihydrolase, arginine arylamidase, and α- and β-glucosidase. Habitat: human digestive tract. Type species: *Kallipyga massiliensis*.

### Description of *Kallipyga massiliensis* *gen. nov.,* sp. nov.

*Kallipyga massiliensis* (mas.il’ien’sis. L. gen. fem. n. *massiliensis*, of Massilia, the Latin name of Marseille where was cultivated strain ph2^T^). It has been isolated from the feces of an obese French patient.

Gram-positive cocci. Strictly anaerobic. Mesophilic. Optimal growth at 37°C. Non-motile and non-sporulating. Colonies are bright grey with 1.0 mm in diameter on blood-enriched Columbia agar. Cells are cocci with a diameter ranging from 0.57 µm to 0.78 µm with a mean diameter of 0.67. Catalase and oxidase activities are negative. Nitrate reduction and indole production are absent. Negative reactions were observed for β-galactosidase, β-galactosidase-6-phosphate, α-arabinosidase, β-glucuronidase, N-acetyl-β-glucosaminidase, glutamic acid decarboxylase, proline arylamidase, leucyl glycine arylamidase, phenylalanine arylamidase, pyroglutamic acid arylamidase, tyrosine arylamidase, alanine arylamidase, glycine arylamidase, histidine arylamidase, glutamyl glutamic acid arylamidase, and serine arylamidase, mannose and raffinose fermentation, esterase, lipase, valine and cystine arylamidase, trypsine, α-chymotrypsine, naphthol-AS-BI-phosphohydrolase, β-galactosidase, β-Glucuronidase, N-acetyl-β-glucosaminidase, α-mannosidase and α-fucosidase. Positive reactions were observed for α-galactosidase, arginine dihydrolase and arginine arylamidase, α and β-glucosidase, esterase lipase, leucine arylamidase and acid phosphatase. Cells weakly oxidized D-ribose, D-glucose, D-fructose and aesculin. Cells are susceptible to amoxicillin, amoxicillin-clavulanic acid, gentamicin 500, penicillin, imipenem, vancomycin, rifampin and nitrofurantoine. The 16S rRNA and genome sequences are deposited in GenBank under accession numbers JN837487 and CAHC00000000, respectively. The G+C content of the genome is 51.40%. The type strain ph2^T^ (= CSUR P241 = DSM 26229) was isolated from the fecal flora of an obese patient in France.
